# Evaluation of a Comprehensive Profile of Salivary Analytes for the Diagnosis of the Equine Gastric Ulcer Syndrome

**DOI:** 10.3390/ani12233261

**Published:** 2022-11-23

**Authors:** Alberto Muñoz-Prieto, José J. Cerón, Camila P. Rubio, María Dolores Contreras-Aguilar, Luis Pardo-Marín, Ignacio Ayala-de la Peña, María Martín-Cuervo, Ida-Marie Holm Henriksen, Julián J. Arense-Gonzalo, Fernando Tecles, Sanni Hansen

**Affiliations:** 1 Interdisciplinary Laboratory of Clinical Analysis of the University of Murcia (Interlab-UMU), Regional Campus of International Excellence ‘Campus Mare Nostrum’, University of Murcia, Campus de Espinardo s/n, Espinardo, 30100 Murcia, Spain; 2 Clinic for Internal Diseases, Faculty of Veterinary Medicine, University of Zagreb, Heinzelova 55, 10000 Zagreb, Croatia; 3 Animal Medicine and Surgery Department, Regional Campus of International Excellence ‘Campus Mare Nostrum’, University of Murcia, Campus de Espinardo s/n, Espinardo, 30100 Murcia, Spain; 4 Animal Medicine, Faculty of Veterinary Medicine of Cáceres, University of Extremadura, Av. de la Universidad S-N, 10005 Cáceres, Spain; 5 Department of Veterinary Clinical Sciences, Veterinary School of Medicine, Sektion Medicine and Surgery, University of Copenhagen, Hoejbakkegaard Allé 5, DK-2630 Høje-Taastrup, Denmark; 6 Institute for Biomedical Research of Murcia, IMIB-Arrixaca, El Palmar, 30120 Murcia, Spain; 7 Division of Preventive Medicine and Public Health Sciences, School of Medicine, University of Murcia, Espinardo, 30100 Murcia, Spain

**Keywords:** equine gastric ulcer syndrome, horse, saliva, biomarkers, diagnosis

## Abstract

**Simple Summary:**

Equine gastric ulcer syndrome (EGUS) is a common and worldwide-distributed clinical situation with highly unspecific clinical signs, which is diagnosed by gastroscopic examination. Saliva is a biological fluid that has been gaining importance over the years as a diagnostic sample. Because previous studies have shown that some biomarkers change their concentration in saliva in horses with EGUS, the possible usefulness of a profile of biomarkers measured in this fluid for EGUS diagnosis has been studied in this report. A total of 23 salivary biomarkers were measured in horses with EGUS, and values were compared with those obtained in healthy animals and horses with diseases with similar symptoms to EGUS but with a negative diagnosis at gastroscopic examination. A total of 17 biomarkers were increased in saliva from horses diagnosed with EGUS compared to healthy animals, and three of those analytes showed a modest but significant statistical power for discriminating EGUS from other diseases.

**Abstract:**

In this report, the measurement of salivary biomarkers as an aid for diagnosis of equine gastric ulcer syndrome (EGUS) was studied. A comprehensive panel of 23 salivary analytes was measured in the saliva of horses affected by EGUS and compared to healthy animals and horses with other diseases clinically similar to EGUS but with a negative diagnosis at gastroscopic examination. A total of 147 horses were included in the study and divided into heathy population (*n* = 12), the EGUS group (*n* = 110), and the group of horses with other diseases (*n* = 25). From the 23 analytes studied, 17 showed increased values in EGUS horses when compared to healthy ones, and uric acid, triglycerides, and calcium were significantly increased in horses with EGUS compared to the group of other diseases. The receiver operating characteristic curve analyses showed a modest but significant discriminatory power of those three analytes to identify EGUS from other diseases with similar symptoms. The discriminatory power enhanced when the results of the three analytes were combined. In conclusion, the results showed that selected salivary analytes could have potential use as biomarkers in horses with EGUS.

## 1. Introduction

Equine gastric ulcer syndrome (EGUS) is a clinical syndrome with a worldwide distribution affecting all breeds regardless of age and sex, although the incidence increases with age [1,2]. There are two different diseases identified in EGUS: the equine squamous gastric disease (ESGD), and the equine glandular gastric disease (EGGD) [3]. The pathogenesis of ESGD involves an acidic attack to the squamous mucosa, with feeding practices and high exercise volume considered as important risk factors [4]. In addition, it can occur in foals with pyloric stenosis due to a delayed gastric emptying [5]. On the other hand, the pathogenesis of EGGD remains unknown, but it is believed to be caused by breakdown of the gastric glandular mucosal defense mechanisms. Stress, infection with bacteria, nonsteroidal anti-inflammatory drugs (NSAIDs), and inhibition of protective prostaglandins have been proposed as possible causes [4,5].

One of the main limitations for EGUS diagnosis is that its clinical signs (such as poor performance, recurrent colic, inappetence, or poor body condition, among others) are nonspecific. In addition, some animals can have gastric lesions without showing any external clinical symptom. Therefore, gastroscopy is the only diagnostic method for EGUS [3]. In this context, the discovery of novel non-invasive diagnostic techniques such as biomarkers, could be a very convenient aid for diagnosis. For medical purposes, a biomarker is defined as a biological observation that can be useful for detection and diagnosis of a concrete clinical situation, monitoring of the clinical course or even predicting the outcome [6]. Traditionally, laboratory biomarkers have been routinely measured in blood, but saliva offers several advantages, such as its non-invasive sampling, which can be performed by non-trained staff, and it can be obtained repeatedly, causing minimum stress to the animals [7].

Recently, some salivary biomarkers have been studied in horses affected by EGUS, such as adenosine deaminase (ADA). This enzyme was increased in horses with ESGD and EGGD compared with healthy ones. In addition, other biomarkers related to oxidative stress, such as ferric-reducing activity of saliva (FRAS), uric acid (UA), and the advanced oxidation protein products (AOPP) were increased in the saliva of horses with EGGD compared with healthy animals, whereas no significant increase was observed in cases of ESGD [8]. Moreover, a proteomic approach comparing saliva of horses with ESGD and EGGD showed that proteins related with epithelial regulation, such as serpin B5 and keratins 15 (KRT15) and 4 (KRT4), were upregulated in ESGD compared to EGGD [9]. Additionally, other salivary biomarkers have been reported to be potentially useful in horses with acute abdominal disease [10,11]. Therefore, the use and possible applications of the sialochemistry, which is defined as a profile of different analytes that can be measured in saliva, is gaining more attention.

The aim of this research was to advance the current knowledge concerning changes that can occur in saliva biomarkers in horses with EGUS and their potential clinical applications. Therefore, in this report differences in salivary biomarkers between healthy horses, horses with EGUS and horses presented with similar clinical signs to EGUS but not diagnosed with that disease were evaluated. For this purpose, a comprehensive panel integrated by 23 analytes included biomarkers related with stress, immune system, and redox status were analyzed in the three groups of animals. Additionally, the possible differences in salivary analytes between the two types of EGUS were explored.

## 2. Materials and Methods

### 2.1. Animals and Diagnosis

All animals included in this study were admitted to the Large Animal Teaching Hospital at the University of Copenhagen between August 2020 and August 2022. Diseased animals included horses with a clinical history of riding issues, weight loss, and changes in temperament and/or pain behaviors, which are clinical signs often compatible with EGUS [3]. Diagnoses were based on different tools including anamnesis, clinical history, physical examination (including weight, body condition score (BCS) based on a nine-point scale, heart rate, respiratory rate, rectal temperature, color mucous membranes, capillary refill time and borborygmus), hematology, and biochemistry. Gastroscopy was performed in all animals after a fasting period of 12 h as previously described [12]. Images obtained by gastroscopy were used for the diagnosis of EGSD and EGGD, according to the ECEIM Consensus Statement [3]. For ESGD diagnosis, an animal was considered positive for this disease when it achieved a score ≤ 1 in the 4-point ESGD gradation scale. Depending on the case, additional diagnostic tools were performed, such as examination per rectum, transabdominal ultrasonography, abdominocentesis, or exploratory laparotomy.

Based on the results of the clinical examinations and diagnostic tests, horses were classified into three different groups.

- EGUS group. The animals showed clinical signs and gastroscopy images compatible with EGUS, according to the criteria previously stated [3]. This group was further stratified into ESGD (and scored by using the 4-point scale indicated above), EGGD or both ESGD and EGGD. Only animals that were diagnosed with EGUS and had no evidence of other diseases were included in this group.
- Horses presenting with clinical signs compatible with EGUS but without gastroscopy images compatible with EGUS. These horses were further diagnosed with another disease or no specific diagnosis was found.
- The healthy population, which was composed of horses admitted for castration or routine health check. Those animals showed no clinical signs of abdominal pain or any other abnormality during physical examination; hematological and biochemical results were within reference values, and had no signs of EGUS after gastroscopy examination.

### 2.2. Sampling

Salivary and blood samplings were performed before performing intravenous sedation and gastroscopy, but immediately after the horses were placed in the examination stock. Saliva samples were obtained as previously reported [10]. A piece of sponge (Esponja Marina, La Griega E. Koronis, Madrid, Spain) of approximately 5.0 × 2.5 × 2.5 cm was introduced into the horse’s mouth until it was soaked with saliva. Immediately after sampling, the sponges were placed in a commercially available device (Salivette, Sarstedt, Aktiengesellschaft & Co., Nümbrecht, Germany). Tubes with saliva were centrifuged at 3000× *g* for 10 min at 4 °C within 30 min of sampling. Saliva was then transferred into Eppendorf tubes and stored at −80 °C until analysis. After saliva sampling, 5 mL of blood were obtained by jugular venipuncture and transferred into tubes (Becton Dickinson Vacutainer Systems Europe) containing ethylenediaminetetraacetic acid (for routine hematology analysis) and clot activator for serum obtention (for routine biochemistry analysis).

Horses were only sampled if guaranteed that they did not receive any feed for at least 12 h. Only saliva with a degree of dirtiness 0 or 1 according to the color scale previously reported (0–4 score) was included [13].

### 2.3. Saliva Biochemistry Profile

The biochemistry profile measured in saliva was integrated by the following parameters:

- Enzymes: adenosine deaminase 1 (ADA1) and 2 (ADA2) isoenzymes, alkaline phosphatase (ALP), aspartate aminotransferase (AST), butyrylcholinesterase (BChE), creatine kinase (CK), γ-glutamyl transferase (gGT), lipase (LIP), lactate dehydrogenase (LDH), and α-amylase (sAA).
- Metabolites and proteins: creatinine (Creat), d-dimer, ferritin (Ferr), total cholesterol (TChol), total proteins (TP), triglycerides (Trig), and urea.
- Redox biomarkers: the advanced oxidation protein products (AOPP), the ferric reducing activity of saliva (FRAS), and uric acid (UA).
- Minerals: total calcium (Ca) and phosphorus (P).

These assays were carried out on an automated chemistry analyzer (Olympus Diagnostica GmbH AU600) by using commercial kits from Beckman (Beckman Coulter Inc., Fullerton, CA, USA) for all assays with the exception of: (a) ADA isoenzymes, which was measured with a Diazyme kit (ADA-D assay kit, Diazyme Laboratories, Poway, CA, USA); (b) BChE, which was measured according to an assay using 5,5′-dithio-bis-(2-nitrobenzoic acid) (DTNB, Sigma Aldrich, Darmstadt, Germany) as chromophore and butyrylthiocholine iodide (BTCI, Sigma Aldrich) as substrate; (c) TP, which was measured by using a commercial colorimetric kit for urine and low-complexity region (LCR) proteins (protein in urine and CSF, Spinreact, Barcelona, Spain); (d) FRAS and AOPP, which were measured by previously published protocols [14,15]. All these assays in saliva have been validated in previous research [8,10,16].

### 2.4. Statistical Analysis

Data was evaluated for normality by using the Shapiro-Wilk test, giving non-parametric distribution in all analytes. Differences between groups (healthy and diseased populations) were assessed by a Kruskal–Wallis test followed by Bonferroni pairwise comparison. Spearman correlation coefficient was calculated between ESGD score and salivary biomarkers in horses with ESGD and healthy horses. A correlation was considered to be strong when the correlation coefficient was ≥0.7. Receiver operating characteristic (ROC) curves were performed for the different analytes in order to know whether they have any value to discriminate the EGUS group from the group of horses with other diseases. Those analytes showing a significant area under the curve (AUC) were selected for calculating cut-off values according to previously described methods [17]. Sensitivity, specificity, and positive and negative likelihood ratios (+LR and −LR, respectively) were calculated from the ROC analyses. All statistical analyses were performed by using a spreadsheet (Excel 2000, Microsoft Corporation, Redmond, WA, USA) and the commercial statistics package SPSS (IBM SPSS Statistics for Windows, Version 28.0. IBM Corp., Armonk, NY, USA). Values of *p* < 0.05 were selected to indicate significance for all analyses. Statistical power of the results (1 − β) obtained from the previous statistical analysis were calculated by a post-hoc analysis by the G-Power program [18] to evaluate if a type I error has been incurred with the number of horses evaluated.

## 3. Results

### 3.1. Animals Included in the Study

A total of 147 horses were included in the study. The healthy group was integrated by 12 animals (5 mares, 7 geldings) of different breeds with a median (interquartile range) age of 6.0 (9.0) years old and BCS of 5.0 (1.0). The EGUS group included 110 animals (42 mares, 68 geldings) of different breeds, with a median age of 11.0 (6.0) years, and a BCS of 6.0 (2.0). This group included 31 diagnosed as having ESGD, 36 with EGGD, and 43 with both ESGD + EGGD. Eighteen of the horses diagnosed with ESGD showed 1 point of severity, 27 showed 2 points, 22 showed 3 points, and 7 showed 4 points of severity. The group with other diseases was composed of 25 animals of diverse breeds (5 mare, 20 geldings) with 10.0 (7.0) years old and BCS of 6.0 (2.0). The final diagnoses of those animals appear in Table S1. No statistically significant differences were detected between groups regarding age and BCS.

### 3.2. Results of the Salivary Biomarkers

**Figure 1:**
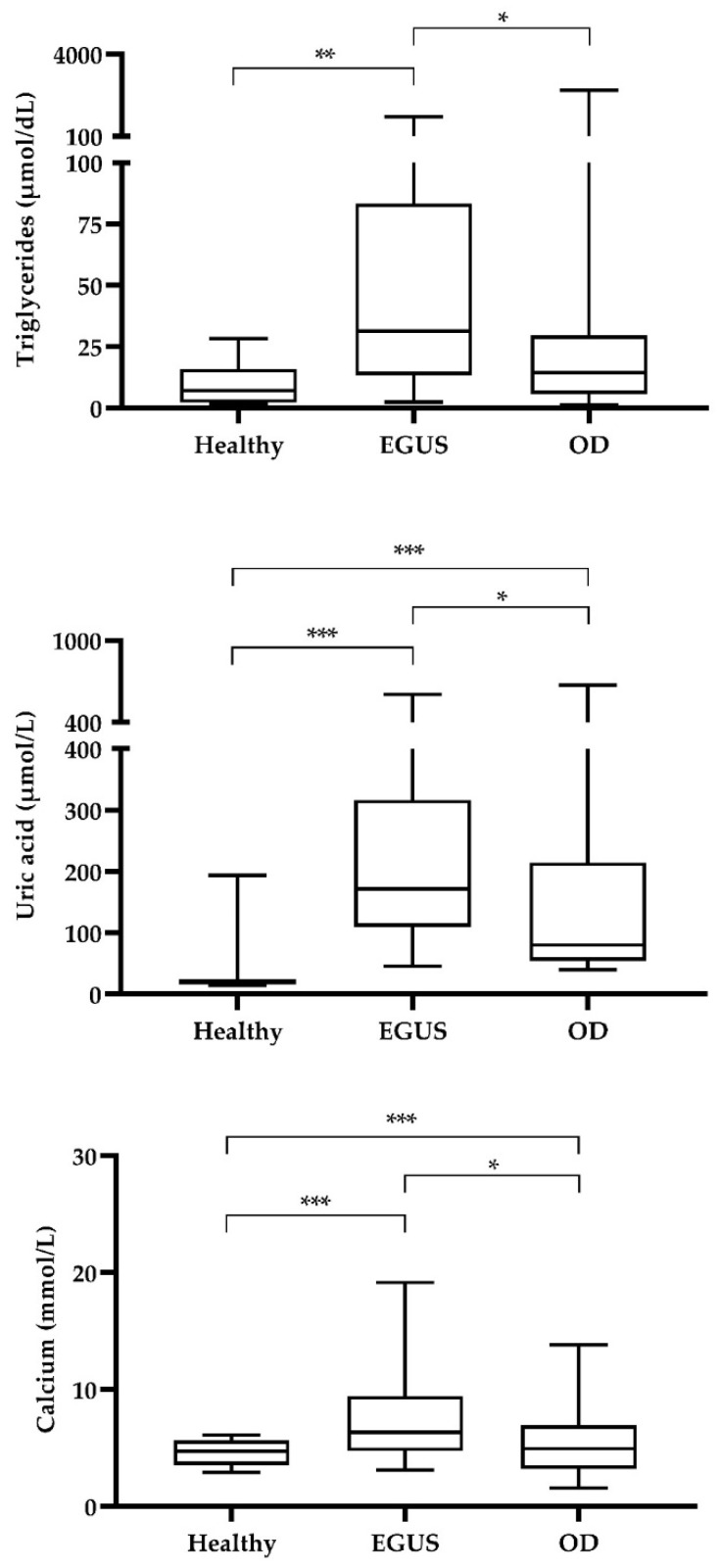
Results obtained of salivary triglycerides, uric acid and calcium in healthy horses (Healthy, *n* = 12), horses diagnosed with EGUS (*n* = 110) and horses diagnosed with other gastrointestinal disorders apart from EGUS but with similar symptoms (OD, *n* = 25). Line shows median value, and box and whiskers show 10–90 percentiles. Statistical analysis: asterisks indicate Bonferroni post-hoc test significant results (*: *p* < 0.05 with H; **: *p* < 0.01 with H; ***: *p* < 0.001).

**Figure 2:**
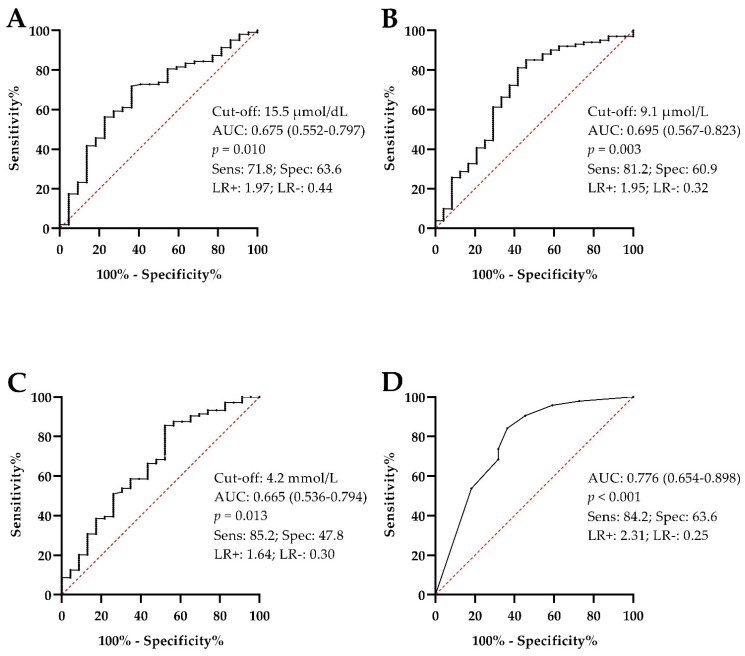
Receiver operating characteristic curves of triglycerides (**A**), uric acid (**B**), calcium (**C**) and the combination of these three biomarkers (**D**) for discriminating EGUS from animals with other diseases with similar clinical symptoms of EGUS but with a negative endoscopy result. AUC, area under the curve; CI, confidence interval; Sens, sensitivity; Spec, specificity; LR, likelihood ratio.

The salivary biomarkers results obtained in the different groups of animals are shown in Table 1. The analytes that showed significantly higher concentrations in the animals with EGUS than in the healthy population were ADA (both isoenzymes), ALP, AST, BChE, CK, gGT, LIP, LDH, sAA, Ferr, TP, Trig, urea, FRAS, UA, Ca, and P (AOPP showed a trend with *p* = 0.055). Many of those biomarkers also showed differences between healthy animals and animals with other diseases, such as ADA isoenzymes, AST, BChE, CK, gGT, LDH, Ferr, TP, urea, UA, and AOPP. The analytes that showed differences between the groups of animals with EGUS and the group of animals with similar clinical signs but with other diseases were Ca, Trig, and UA, which showed higher values in the EGUS group (Table 1 and Figure 1), and Ferr that were higher in the horses with other diseases. When ROC analyses were performed to assess the discriminatory power of the analytes that showed increases in horses diagnosed with EGUS compared to horses with similar clinical signs but with no EGUS, Trig, UA, and Ca showed a modest but significant AUC (Figure 2).

The values obtained in animals with EGUS when divided in those with ESGD, EGGD, and both ESGD + EGGD are shown in Table S2. In general, no differences were seen between animals with ESGD, EGGD, or both ESGD + EGGD, with the exceptions of ADA2, which was higher in the ESGD + EGGD group compared with ESGD only, and Trig and Ca that were higher in the ESGD + EGGD group compared with the horses with EGGD only.

Table 2 shows the correlation coefficients between ESGD grade and salivary biomarkers. Strong correlations (Spearman correlation coefficients higher than 0.7) were observed with ADA1, ADA2, UA, and AST.

## 4. Discussion

In this research, the potential use of a panel of salivary biomarkers to detect EGUS in horses was evaluated. The criteria for the selected biomarkers included in our study was the following. ADA, FRAS, AOPP, and uric acid were chosen because they have previously shown changes in horses with EGUS [8]. In addition, gGT, CK, urea, TP, P, and sAA were in the profiles because they were reported to change in equine diseases such as colic [10,11]. Finally, other analytes related to metabolism and the function of different tissues and organs, which are included in the concept of sialochemistry, such as AST, ALP, LIP, LDH, BChE, d-dimer, Creat, TChol, Trig, P, and Ca, were also evaluated. These analytes have the advantage of being able to be adapted to automated analyzers and therefore could be used for routine clinical purposes. Overall, these analytes constitute a comprehensive profile involving biomarkers related to stress, immune system and redox status [7].

Different mechanisms could explain the presence of the biomarkers in saliva. For example, free cortisol passes from blood to saliva by passive diffusion of the molecule to the salivary gland, and therefore saliva reflects the circulating levels of the molecule [19]. On the other hand, other biomarkers are directly produced by the salivary glands, such as sAA, which is synthesized after stimulation of the glands by the activation of the automated nervous system [20]. In cases such as ADA, the source of the biomarker in saliva is unknown and it does not correlate with serum [16].

In our report, we found 17 analytes that showed higher values in EGUS compared to healthy animals. When the ESGD animals with grade 1 were excluded to the statistical analyses (since a grade 1 ESGD could not be considered clinically relevant by some clinicians) similar results were achieved, with the exception that ADA2 and Ca did not show any differences between the horses with the different types of EGUS (data not shown). From these 17 analytes, we will discuss ADA, ALP, AST, CK, gGT, and FRAS because of their physiopathological interest and clinical significance, or because they showed changes of higher magnitude when compared with the healthy animals.

ADA is an enzyme group related with the function of the lymphoid system [21]. In human patients with gastric ulcer, ADA showed higher values in the mucosa close to the ulcer crater and decreased when treatment was successful. Therefore, a possible role in peptic ulcer healing was proposed [22]. In a previous report from our group, ADA was increased in saliva of horses with EGUS. Based on the results observed in this research, it could be postulated that horses with EGUS are likely to present increasing values of salivary ADA1 and ADA2. In addition, a strong correlation was observed between ESGD grade and salivary levels of ADA isoenzymes, a fact that should be further studied in order to know whether salivary ADA could potentially be a reliable indicator of the severity of this process. It should be pointed out that ADA can increase in other diseases, and for example, in horses with colic of intestinal etiology ADA1 was higher in non-survivors than in survivors [8].

In our study, an increase of ALP in saliva samples was found in EGUS-affected horses compared to healthy ones, but not in horses affected by other diseases. ALP is an enzyme mainly found in the bone and liver, but it has also been detected in the intestines of horses [23,24]. Serum activity of this enzyme has been associated with the severity of gastric ulcers in horses [25]. ALP values can rise because of increased bone turnover or liver expression as a consequence of intense training and increased concentrate feed administration, respectively, which are two predisposing factors to gastric ulcers.

AST is a transaminase enzyme present in several tissues that has an important role in amino acid metabolism. In humans, serum elevations of this enzyme are mainly found related to liver, heart, or skeletal muscle damage [26]. However, research has shown the presence of transaminases in the human gastric mucous membrane and that AST are secreted into the gastric juice, particularly after damage to the stomach wall [27]. In fact, an in vitro approach performed with a guinea pig’s gastric glands showed the release of AST after incubation with clinical isolates of *Helicobacter pylori* due to the cytotoxic activity of these bacteria over the gastric mucosal cells [28]. Further studies should be performed to elucidate the mechanism of the increase of AST in saliva in the affected horses included in the present study.

CK is an enzyme that catalyzes the phosphorylation of adenosine triphosphate and creatine to adenosine diphosphate and phosphocreatine, playing an important role in the regulation of cellular energy metabolism [29]. An increase in serum CK was found in a murine model of gastric ulcers induced by stress related to water immersion and restraint [30]. This model produces oxidative stress in several tissues, including stomach, and those authors concluded that CK could be released directly from gastric mucosa of ulcers into the bloodstream, because it is expressed in gastric mucosa [30]. A positive correlation was reported between serum and salivary CK activities after muscle damage in dogs [31] and horses [32]. Although CK levels found in saliva could reflect an increase in serum levels, it was postulated that in horses it seems that CK could be released directly by the salivary gland and that this mechanism could be more important than linkage from serum [32]. Due to the aforementioned reasons, the diagnostic meaning of increased CK in saliva should be interpreted cautiously.

gGT is an enzyme involved in the glutathione (GSH) metabolism and it can be produced by multiple organs in the body, including the pancreas, seminal vesicles, kidneys, biliary tract, and liver [33]. In humans, there are two possible links between gGT and stomach disease. First, the increased serum gGT was reported in response to oxidative stress in the gastrointestinal system. This gGT can produce additional alterations because it participates in catabolism of extracellular GSH, which induces the production of reactive oxygen species (ROS) by thiol-dependent iron reduction [34]. Therefore, the persistent production of ROS by increased gGT activity may cause even tumor progression in the gastrointestinal tract [35]. Secondly, this enzyme is involved in the pathogenesis of gastric disease induced by *H. pylori*, inducing the production of hydrogen peroxide (H_2_O_2_) and increasing the risk of oxidant-related gastric epithelial injury [36]. *H. pylori* has not been shown to cause ulcers in horses, although it has been isolated from the squamous and glandular mucosa of horses. Other resident bacteria such as *Escherichia coli*, *Lactobacillus* spp., and *Streptococcus* spp. are suspected to contribute to the worsening of squamous ulcers [37], although it is not known whether gGT could have a similar role in those pathogens.

FRAS determines the total antioxidant capacity of saliva, and measures the ability of the non-enzymatic antioxidants present in saliva to reduce ferric-tripyridyltriazine to the ferrous form [14]. In a pilot study performed in horses with gastric ulcers, the salivary levels of FRAS were increased in horses with EGGD compared with healthy ones; thus, the involvement of oxidative stress in the pathogenesis of this disease was postulated [8]. Similarly, in our report FRAS was increased in horses with EGGD and ESGD + EGGD, but not in ESGD horses.

UA, Trig, and Ca were the analytes that showed significantly higher values in horses with EGUS than in the group with similar clinical signs but other diseases. For this reason, these analytes were further studied and ROC curve analyses were made, resulting that UA, Trig, and Ca showed significant, though modest, AUC values for discriminating EGUS-diagnosed horses from those presented with other diseases. When those analytes were combined, the discriminating power enhanced. Recent examples exist about the increased diagnostic power of the combination or two or more biomarkers [38,39].

UA is a molecule with antioxidant activity, but an excess of this molecule induces cytokine and chemokine production, enhancing inflammation and causing endothelial dysfunction and fibrosis [40]. In our report, UA was the analyte that showed higher discriminatory power between EGUS animals and horses with clinical signs and without ulcers. In humans, higher UA serum levels were reported in patients with ulcers than in healthy individuals and those with gastritis but without ulcers, with the increases in serum probably due to its release from damaged gastric cells into the bloodstream [41]. Moreover, in horses, serum UA levels have been reported to be useful in discriminating between horses with EGUS and horses with other diseases of intestinal origin [8]. Although no significant differences in UA between ESGD and EGGD were found in this study, values in EGGD were higher than in ESGD, in line with a previous report, that could indicate that in general there is a higher damage in gastric cells in EGGD compared to ESGD [8].

Trig was higher in a group of people positive for *H. pylori* than the group that was negative. In addition, within the group of people positive for *H. pylori*, the level of Trig was higher in people with gastric mucosal erosion, gastric ulcer, and duodenal ulcer than that in people with normal gastric mucosa or mild gastritis [42]. In another human report, no association was found between Trig values and infection by *H. pylori*, although the presence or absence of gastric ulcers was not established in this study [43]. Horses with gastric ulcers may be reluctant to eat food as it causes pain, which could mobilize Trig from fat to produce energy. Anyway, the possible influence of gastric ulcers on Trig levels should be further studied.

Ca is involved in the secretion of gastrin by the stomach G cells [44], leading to increased gastrin serum levels in patients with hypercalcemia [45]. Intravenous Ca administrations are used for the diagnosis of gastrinoma in human patients, because the excess of Ca induces the release of huge amounts of gastrin by the tumor. The excess of gastrin has been related with the appearance of gastric ulcers in humans because this hormone induces the release of hydrochloric acid (HCl) by the parietal gastric cells [46]. In fact, ulcers are one of the main consequences of Zollinger-Ellison syndrome, in which excess of gastrin secretion is produced [47]. In horses, it has been proven that exercise has an effect upon the gastric hormonal response to a meal, producing an increased post-feeding gastrin values [48]. Although it is not known whether this elevated gastrin contributes to ulceration in horses, a high exercise volume is recognized to be an important risk factor for EGUS [4]. In humans, it was established that recurrence of peptic ulcer disease is accompanied by a significant increase of Ca in blood [49], a fact that could explain the higher Ca levels in horses with EGUS than in the horses with other diseases found in this study. In spite of this, it is important to take into account that several factors could increase Ca levels, such as chronic renal failure, neoplasia or hyperparathyroidism [50]. This could be the reason why in the ROC study the specificity of this analyte was lower than its sensitivity.

This report had several limitations. One was the small number of healthy animals included; from an ethical point of view, it is difficult to justify a gastroscopic examination in clinically healthy animals. In addition, the number of horses in the group with diseases different than EGUS is low. These facts, together with the great inter-individual variability observed in the results (high interquartile range values), could compromise the statistic results. Due to these reasons, this report should be considered as a pilot approach, and larger studies should be made in which the diagnostic performance of the analytes showing the potential to diagnose EGUS should be evaluated. In spite of this, the statistical power obtained in this report was higher than 0.8, indicating that from a statistical point of view, the number of animals included in this study was adequate. Another limitation is that because animals were sampled as they arrived at the hospital, the possible influence due to circadian variations was not considered. This could be important for some analytes such as ALP, Ca, and P, because their serum levels have been related to circadian variations [51] and also changes have been described in analytes such as ADA, BChE, or CK in saliva [52]. In addition, samples were taken after receiving the animal in the hospital, and therefore transportation and restraining of the animals in a stock could have influenced stress-related biomarkers. Although this management was similar for all animals and, therefore, this type of bias was presumably equal between the groups. Moreover, although in the diagnostic work-up the horses with EGUS did not have any other evident concomitant disease, this could not be completely excluded. Overall, most of the saliva biomarkers can significantly increase in other diseases apart from EGUS, being unspecific to detect this disease. In this line, it is important to point out that these analyses may be used to screen horses that should undergo gastroscopy, rather than basing a diagnosis just on the concentrations of analytes in saliva, and therefore these analytes should not replace gastroscopy in any case.

## 5. Conclusions

In conclusion, this study demonstrated that 17 salivary analytes (ADA1, ADA2, ALP, AST, BChE, CK, gGT, LDH, sAA, Ferr, TP, Trig, urea, FRAS, UA, Ca, and P) were elevated in horses with EGUS compared with healthy horses. From those 17 analytes, UA, Trig, and Ca could have a significant discriminant power between horses with EGUS compared to horses with other diseases with similar clinical signs but without ulcers. These analytes could have potential use as biomarkers in horses with EGUS. For example, an ADA value within the range of healthy horses in our study could indicate that the horse is not likely to have EGUS at gastroscopy. In addition, higher values of UA, Trig, and Ca in a horse with clinical signs of EGUS would indicate a high probability of having EGUS at gastroscopy. These assays have the advantages of being non-invasive and also easy to measure because most of them are commercially available and are often included in the routine biochemistry profiles in clinical pathology laboratories. Further research using a larger population of horses will be needed to confirm these findings and the potential practical application of these salivary analytes in the diagnosis and treatment monitoring of EGUS.

## Figures and Tables

**Table 1:** Results of salivary analytes in healthy horses (*n* = 12), horses diagnosed with EGUS (*n* = 110) and horses diagnosed with other gastrointestinal disorders apart from EGUS but with similar symptoms (OD, *n* = 25). Median (interquartile range) are expressed. Statistical analysis: *p* value indicates Kruskal–Wallis test result; asterisks indicate Bonferroni post-hoc test significant results with H group (*: *p* < 0.05 with H; **: *p* < 0.01 with H; ***: *p* < 0.001); letters indicate Bonferroni post-hoc test significant results with the OD group (a: *p* < 0.05); 1 − β: statistical power.

	Healthy	EGUS	OD	*p* Value	1 − β	Size Effect
Enzymes						
ADA1 (IU/L)	17.7 (21.7)	170.8 (170.3) ***	103.7 (282.1) **	<0.001	1.00	1.01
ADA2 (IU/L)	0.4 (0.6)	4.2 (6.5) ***	3.1 (7.1) ***	<0.001	1.00	0.95
ALP (IU/L)	49.5 (71.8)	130.0 (280.8) **	104.8 (214.4)	0.004	0.99	0.46
AST (IU/L)	48.1 (28.5)	290.4 (455.8) ***	201.2 (731.3) ***	<0.001	1.00	0.97
BChE (IU/mL)	6.0 (5.4)	40.4 (52.1) ***	48.0 (76.3) ***	<0.001	1.00	1.02
CK (IU/L)	3.9 (4.6)	36.7 (58.6) ***	27.2 (55.7) ***	<0.001	1.00	0.99
gGT (IU/L)	9.8 (15.8)	95.5 (130.2) ***	64.2 (161.7) **	<0.001	1.00	0.98
LIP (IU/L)	40.5 (19.4)	43.9 (45.5)	83.1 (145.3)	0.222	0.90	0.24
LDH (IU/L)	171.0 (221.9)	1102.4 (1443.5) ***	714.0 (1873.4) **	<0.001	1.00	0.70
sAA (IU/L)	4.3 (2.7)	6.8 (19.2) **	7.2 (5.8)	0.011	0.98	0.40
Metabolites and proteins						
Creat (µmol/L)	13.3 (11.5)	17.7 (24.6)	17.7 (24.6)	0.249	0.93	0.23
d-dimer (µg/mL)	0.2 (0.1)	0.5 (2.2)	0.5 (1.6)	0.175	0.94	0.29
Ferr (pmol/L)	19.6 (32.6)	41.8 (25.4) ** a	52.8 (45.2) ***	<0.001	0.98	0.42
TChol (µmol/L)	70.2 (2.6)	70.2 (15.6)	70.2 (2.6)	0.245	0.91	0.13
TP (mg/dL)	50.5 (55.4)	471.3 (703.8) ***	269.2 (612.6) **	<0.001	1.00	0.80
Trig (µmol/dL)	7.2 (13.8)	31.2 (69.9) ** a	14.4 (23.9)	<0.001	0.99	0.49
Urea (mmol/L)	2.7 (3.2)	5.6 (7.1) *	7.5 (9.7) **	0.007	0.99	0.49
Redox biomarkers						
AOPP (µmol/L)	94.0 (181.4)	222.8 (307.2)	230.0 (322.3) *	0.032	0.99	0.44
FRAS (µmol/L)	226.7 (487.0)	656.0 (590.0) **	452.0 (833.2)	0.002	0.96	0.34
UA (µmol/L)	21.2 (7.1)	171.7 (207.1) ***a	80.2 (15.9) **	<0.001	1.00	0.90
Minerals						
Ca (mmol/L)	4.8 (2.1)	6.4 (4.7) * a	5.0 (3.8)	0.002	0.99	0.41
P (mmol/L)	0.2 (0.1)	0.3 (0.5) **	0.3 (0.2)	0.007	0.97	0.36

ADA1, adenosine deaminase isoenzyme 1; ADA2, adenosine deaminase isoenzyme 2; ALP, alkaline phosphatase; AST, aspartate aminotransferase; BChE, butyrylcholinesterase; CK, creatine kinase; gGT, γ-glutamyl transferase; LIP, lipase; LDH, lactate dehydrogenase; sAA, α-amylase (sAA); Creat, creatinine; Ferr, ferritin; TChol, total cholesterol; TP, total protein; Trig, triglycerides; AOPP, advanced oxidation protein products; FRAS, ferric reducing activity of saliva; UA, uric acid; Ca, calcium; P, phosphorus.

**Table 2:** Spearman correlation coefficients obtained between ESGD score and the salivary biomarkers. Animals from the ESGD group (*n* = 31) and the healthy group (*n* = 12) were included in this study. Statistical analysis: *: *p* < 0.05; **: *p* < 0.01.

Enzymes		Metabolites and Proteins		Redox Biomarkers	
ADA1:	0.759 **	Creat:	0.385*	AOPP:	0.257
ADA2:	0.745 **	d-dimer:	0.341*	FRAS:	0.493 **
ALP:	0.519 **	Ferr:	0.600 **	UA:	0.703 **
AST:	0.734 **	TChol:	0.243		
BChE:	0.647 **	TP:	0.623 **	Minerals	
CK:	0.602 **	Trig:	0.240	Ca:	0.410 *
gGT:	0.682 **	Urea:	0.450 **	P:	0.340 *
LIP:	0.555 **				
LDH:	0.597 **				
sAA:	0.304				

ADA1, adenosine deaminase isoenzyme 1; ADA2, adenosine deaminase isoenzyme 2; ALP, alkaline phosphatase; AST, aspartate aminotransferase; BChE, butyrylcholinesterase; CK, creatine kinase; gGT, γ-glutamyl transferase; LIP, lipase; LDH, lactate dehydrogenase; sAA, α-amylase (sAA); Creat, creatinine; Ferr, ferritin; TChol, total cholesterol; TP, total protein; Trig, triglycerides; AOPP, advanced oxidation protein products; FRAS, ferric reducing activity of saliva; UA, uric acid; Ca, calcium; P, phosphorus.

## Data Availability

The data presented in this study are available on request from the corresponding author.

## Supplementary Materials

The following supporting information can be downloaded at: https://www.mdpi.com/article/10.3390/ani12233261/s1, Table S1: Final diagnoses and reason for gastroscopy of the 25 animals suspected for Equine Gastric Ulcer Disease but with negative result after gastroscopy; Table S2: Results of salivary analytes in saliva of horses with the Equine Squamous Gastric Disease (ESGD, *n* = 31), and the Equine Glandular Gastric Disease (EGGD, *n* = 33), or having both (ESGD + EGGD, *n* = 43).
