# Supplementary material for: Evaluation of a Comprehensive Profile of Salivary Analytes for the Diagnosis of the Equine Gastric Ulcer Syndrome
Source: Animals (Basel). 2022 Nov 23;12(23):3261. doi: 10.3390/ani12233261 (PMC9740180; doi:10.3390/ani12233261)
Supplement: Supplementary file 1 [file animals-12-03261-s001.zip › animals-2001756-supplementary.pdf]

Table S1. Final diagnoses and reason for gastroscopy of the 25 animals suspected for Equine Gastric Ulcer Disease but with negative result after gastroscopy.

Final diagnosis	Reason for gastroscopy
Epiploic foramen entrapment of small intestine, surgically corrected	Epiploic foramen hernia surgery
Unspecific colic, euthanized with no findings on autopsy	Colic and fever
Behavioural issues, found to be due to severe cataract	Behavioural change
Head shaking and impaction	Colic
Unspecific colic	Colic
Enteritis	Colic
Unspecific colic after fracture surgery	Diarrhea, stressed after orthopedic surgery
Left dorsal displacement and impaction	Colic
Stress induced diarrhea, rule out other causes	Loose feaces
Gastric impaction	Recurrent colic
Dysbiosis, transfaunation as treatment	Colic
Chronic eosinophilic enteritis, found during necropsy	Fever
Flexura pelvic impaction	Colic
Sand and inflammatory bowel disease	Colic
Right dorsal displacement	Colic
Eosinophilic duodenitis	Poor appetite
Upper airway viral infection	Control
Sand accumulation in colon	Riding issues
Chronic kidney failure, euthanized months later	Chronic kidney failure
Sand accumulation in colon	Colic
Unspecific colic	Colic
Fecal dysbiosis	Chronic diarrhea
Colic, suspected due to adherences in the abdomen	Colic
Behavioural issues, no diagnosis	Riding issues
Mild equine asthma	Poor performance

Table S2. Results of salivary analytes in saliva of horses with the Equine Squamous Gastric Disease (ESGD, n = 31), and the Equine Glandular Gastric Disease (EGGD, n = 33), or having both (ESGD+EGGD, n = 43). Median (interquartile range) are expressed. Statistical analysis: *p* value indicates Kruskal-Wallis test result; letters indicate Bonferroni post-hoc test significant results with the OD group (a: $p < 0.05$ with ESGD group; b: $p < 0.01$ with EGGD group).

	ESGD	EGGD	ESGD + EGGD	*p* Value
Enzymes				
ADA1 (IU/L)	129.5 (146.5)	166.9 (175.5)	191.6 (246.9)	0.095
ADA2 (IU/L)	3.0 (5.5)	4.2 (5.5)	4.9 (9.1)a	0.026
ALP (IU/L)	129.6 (241.6)	128.7 (147.0)	173.6 (388.0)	0.327
AST (IU/L)	275.0 (382.3)	290.4 (335.9)	298.4 (466.4)	0.576
BChE (IU/mL)	36.0 (65.2)	41.7 (42.3)	41.2 (58.4)	0.799
CK (IU/L)	32.8 (37.9)	31.2 (59.4)	45.1 (65.7)	0.164
gGT (IU/L)	78.8 (148.8)	91.5 (103.9)	106.4 (181.6)	0.443
LIP (IU/L)	41.2 (57.1)	28.8 (44.4)	51.9 (33.9)	0.057
LDH (IU/L)	840.8 (1324.8)	880.0 (1160.0)	1473.2 (2211.6)	0.068
sAA (IU/L)	6.1 (43.8)	6.8 (11.9)	7.5 (21.7)	0.672
Metabolites and proteins				
Creat (μmol/L)	17.7 (24.8)	18.6 (17.7)	17.7 (35.4)	0.936
d−dimer (μg/mL)	0.4 (2.9)	0.4 (1.5)	0.8 (2.5)	0.471
Ferr (pmol/L)	41.3 (29.0)	49.0 (28.1)	41.6 (30.1)	0.598
TChol (μmol/L)	70.2 (75.4)	70.2 (10.4)	70.2 (7.8)	0.493
TP (mg/dL)	509.8 (740.2)	326.6 (381.6)	519.4 (878.0)	0.305
Trig (μmol/dL)	32.8 (66.8)	16.5 (34.3)	50.2 (124.7)b	0.001
Urea (mmol/L)	8.9 (7.3)	5.6 (5.2)	5.3 (9.3)	0.275
Redox biomarkers				
AOPP (μmol/L)	216.4 (332.4)	276.8 (297.8)	191.4 (305.0)	0.639
FRAS (μmol/L)	572.8 (901.1)	680.5 (401.6)	752.9 (582.0)	0.679
UA (μmol/L)	149.9 (318.6)	176.4 (163.4)	216.5 (210.6)	0.607
Minerals				
Ca (mmol/L)	6.3 (7.2)	5.4 (2.8)	7.5 (4.3)b	0.011
P (mmol/L)	0.2 (0.5)	0.3 (0.2)	0.4 (0.4)	0.569

ADA1: adenosine deaminase isoenzyme 1; ADA2: adenosine deaminase isoenzyme 2; ALP: alkaline phosphatase; AST: aspartate aminotransferase; BChE: butyrylcholinesterase; CK: creatine kinase; gGT: γ−glutamyl transferase; LIP: lipase; LDH: lactate dehydrogenase; sAA: α−amylase; Creat: creatinine; Ferr: ferritin; TChol: total cholesterol; TP: total protein; Trig: triglycerides; AOPP: advanced oxidation protein products; FRAS: ferric reducing activity of saliva; UA: uric acid; Ca: calcium; P: phosphorus.
